# Identification and Validation of a Ferroptosis-Related Long Non-coding RNA Signature for Predicting the Outcome of Lung Adenocarcinoma

**DOI:** 10.3389/fgene.2021.690509

**Published:** 2021-07-22

**Authors:** Zhiyuan Zheng, Qian Zhang, Wei Wu, Yan Xue, Shuhan Liu, Qiaoqian Chen, Donghong Lin

**Affiliations:** Medical Technology and Engineering College of Fujian Medical University, Fuzhou, China

**Keywords:** ferroptosis, lung adenocarcinoma, long non-coding RNA signature, prognosis, The Cancer Genome Atlas

## Abstract

**Background:**

Ferroptosis is a recently recognized type of programmed cell death that is involved in the biological processes of various cancers. However, the mechanism of ferroptosis in lung adenocarcinoma (LUAD) remains unclear. This study aimed to determine the role of ferroptosis-associated long non-coding RNAs (lncRNAs) in LUAD and to establish a prognostic model.

**Methods:**

We downloaded ferroptosis-related genes from the FerrDb database and RNA sequencing data and clinicopathological characteristics from The Cancer Genome Atlas. We randomly divided the data into training and validation sets. Ferroptosis-associated lncRNA signatures with the lowest Akaike information criteria were determined using COX regression analysis and the least absolute shrinkage and selection operator. The risk scores of ferroptosis-related lncRNAs were calculated, and patients with LUAD were assigned to high- and low-risk groups based on the median risk score. The prognostic value of the risk scores was evaluated using Kaplan–Meier curves, Cox regression analyses, and nomograms. We then explored relationships between ferroptosis-related lncRNAs and the immune response using gene set enrichment analysis (GSEA).

**Results:**

Ten ferroptosis-related lncRNA signatures were identified in the training group, and Kaplan–Meier and Cox regression analyses confirmed that the risk scores were independent predictors of LUAD outcome in the training and validation sets (all *P* < 0.05). The area under the curve confirmed that the signatures could determine the prognosis of LUAD. The predictive accuracy of the established nomogram model was verified using the concordance index and calibration curve. The GSEA showed that the 10 ferroptosis-related lncRNAs might be associated with tumor immune response.

**Conclusion:**

We established a novel signature involving 10 ferroptosis-related lncRNAs (LINC01843, MIR193BHG, AC091185.1, AC027031.2, AL021707.2, AL031667.3, AL606834.1, AC026355.1, AC124045.1, and AC025048.4) that can accurately predict the outcome of LUAD and are associated with the immune response. This will provide new insights into the development of new therapies for LUAD.

## Introduction

Lung cancer is a common type of malignancy worldwide (Sung et al., 2021) and is also the most lethal. It comprises small cell and non-small cell lung cancer types (Garon et al., 2021). Lung adenocarcinoma (LUAD) is the primary type of NSCLC; it is more frequent than squamous cell lung cancer, thus being the most common histological subtype of primary lung cancer (Lortet-Tieulent et al., 2014). Due to the lack of specific techniques that could diagnose cancer in the early stages (Goulart et al., 2021), more useful prognostic biomarkers and therapeutic targets are required.

Ferroptosis is a recently established type of programmed cell death that is iron dependent and characterized by the accumulation of intracellular reactive oxygen species (Dixon et al., 2012). It differs from apoptosis, necrosis, and autophagy in terms of morphology, biochemistry, and genetics (Chen X. et al., 2021). Ferroptosis plays a key role in killing tumor cells and inhibiting tumor growth (Jiang et al., 2021; Wang H. et al., 2021). Accumulating evidence indicates that ferroptosis is involved in the biological processes of LUAD (Wohlhieter et al., 2020; Li et al., 2021; Lou et al., 2021). However, the regulation of ferroptosis remains obscure, and it is far from being applied to cancer therapy. Therefore, identifying the key regulators of ferroptosis is a key step in broadening the options for treating cancer.

Long non-coding RNAs (lncRNAs) are transcripts of > 200 nucleotides that typically do not encode proteins (Kopp and Mendell, 2018). lncRNAs play important roles in many processes, such as epigenetic regulation, cell cycle regulation, and cell differentiation regulation by mediating transcriptional activation, interference, and chromosomal modification (Pang et al., 2019). In particular, the abnormal expression or function of lncRNAs might be associated with various diseases, including cancer. lncRNAs may affect ferroptosis; for example, the nuclear lncRNA LINC00618 accelerates ferroptosis in a manner dependent upon apoptosis in leukemia (Wang Z. et al., 2021). The lncRNA PVT1 regulates ferroptosis through miR-214-mediated TFR1 and p53 in patients with acute ischemic stroke (Lu et al., 2020). The lncRNAs GABPB1-AS1 and GABPB1 regulate oxidative stress during erastin-induced ferroptosis in HepG2 hepatocellular carcinoma cells (Qi et al., 2019). The lncRNA LINC00336 inhibits ferroptosis in lung cancer by functioning as a competitive endogenous RNA (Wang M. et al., 2019). The lncRNA MT1DP increases non-small cell lung cancer sensitivity to ferroptosis through regulating the miR-365a-3p/NRF2 axis (Meng et al., 2020). Therefore, ferroptosis-related lncRNAs involved in predicting the outcome of LUAD should be determined to provide a theoretical basis for novel strategies to treat patients with LUAD.

Previous studies have shown that one mechanism for non-coding RNA to participate in gene regulatory networks is through the direct binding of lncRNA to other RNA molecules to regulate their stability or translation. These interactions rely on the binding of lncRNA to its RNA target and either creating a substrate for protein function or inhibitory protein effectors (Anastasiadou et al., 2018). Therefore, we use the limma package in R, one of the high-throughput analysis methods, to conduct a comprehensive analysis of the genome of LUAD (Ge et al., 2020). Through the co-expression analysis, the least absolute shrinkage and selection operator (lasso) regression, and COX regression (Wang et al., 2013; Hu et al., 2014), we identified ferroptosis-related lncRNAs that are associated with the prognosis of LUAD, constructed a nomogram with which to determine the prognosis of LUAD, and herein discuss the relevance of the model to predicting the immune response. Our findings should help to improve the early diagnosis rate of LUAD and provide a theoretical basis for precise, individualized treatment.

## Materials and Methods

### Acquisition of Gene Expression and Clinical Data

Gene expression profiles and clinical data from patients with LUAD were downloaded from The Cancer Genome Atlas (TCGA). mRNAs and lncRNAs were encoded according to GENCODE Release 29 (GRCh38.p12). We collected clinical data including sex, age, and TNM, pT, pN, and pM stages as well as follow-up data. We excluded patients with incomplete clinicopathological characteristics. Considering the possibility of non-cancer-death, we also excluded patients who survived ≤ 30 days. We finally included data from 443 patients and randomly assigned them to training (*n* = 223) and validation (*n* = 220) sets using the caret package in R.

### Ferroptosis-Associated lncRNAs

We obtained 259 ferroptosis-associated gene sets from FerrDb, the first manually curated resource for regulators and markers of ferroptosis that was released in December 2019 (Zhou and Bao, 2020). Ferroptosis-associated lncRNAs detected using the limma package in R were identified by correlation analysis between ferroptosis-related genes and lncRNA expression levels in the LUAD samples. We determined the Pearson correlation coefficients, and the threshold was established as a correlation coefficient > 0.4 and *P* < 0.001.

### Establishment of Prognostic Ferroptosis-Associated lncRNA Signatures

We screened lncRNAs associated with the overall survival (OS) of patients with LUAD using univariate Cox regression analysis. Further screening was based on lasso regression to avoid overfitting, and we adjusted the L1 penalty parameter through 10-fold cross-validation to reduce the number of genes. Genes with a repetition frequency > 900 in 1,000 replicates were considered to be more closely associated with OS. We established prognostic lncRNA signatures using multivariate Cox regression and then constructed a lncRNA signature with the lowest Akaike information criterion (AIC) (Susko and Roger, 2020). The risk score for each patient with LUAD was calculated based on the amount of lncRNA expression and the corresponding coefficient as:

β⁢l⁢n⁢c⁢R⁢N⁢A⁢1×E⁢x⁢p⁢r⁢e⁢s⁢s⁢i⁢o⁢n⁢l⁢n⁢c⁢R⁢N⁢A⁢1+β⁢l⁢n⁢c⁢R⁢N⁢A⁢2×ExpressionlncRNA2+…+β⁢lncRNAn×ExpressionlncRNAn

### Evaluation of Risk Score Prediction Ability and Nomogram Construction

Patients in the training and validation sets were assigned to high- and low-risk groups using the median risk score of the training group. Kaplan–Meier survival curves were constructed, and predictive performance was evaluated using receiver operating characteristics (ROC) curves. We conducted t-distributed stochastic neighbor embedding (t-SNE) using the Rtsne package in R to reduce the dimensions and visualize ferroptosis status based on the high- and low-risk groups. We applied independent prognostic factors determined *via* multivariate Cox regression to construct a prognostic nomogram using the rms package in R. The accuracy of the nomogram was verified using the Harrell concordance (*C*) index and a calibration curve.

### Construction of a Ferroptosis-Related lncRNA–mRNA Co-expression Network

Correlations between co-expressed lncRNAs and mRNAs associated with ferroptosis were analyzed using a co-expression network and a Sankey diagram visualized using Cytoscape and the ggalluvial package in R.

### Gene Set Enrichment Analysis

Potential immune responses between the high- and low-risk patients were investigated using gene set enrichment analysis (GSEA) (Subramanian et al., 2005), and *P* < 0.05, with a false discovery rate (FDR *q*) < 0.05, was considered statistically significant.

### Statistical Analysis

Clinicopathological characteristics were compared between the training and validation sets using chi-square tests, and relationships between clinicopathological characteristics and lncRNA expression were compared using Mann–Whitney *U*-tests. All data were statistically analyzed using R (v. 4.0.1), and statistical significance was set at *P* < 0.05.

## Results

### Acquisition of lncRNA Associated With Ferroptosis

**FIGURE 1:**
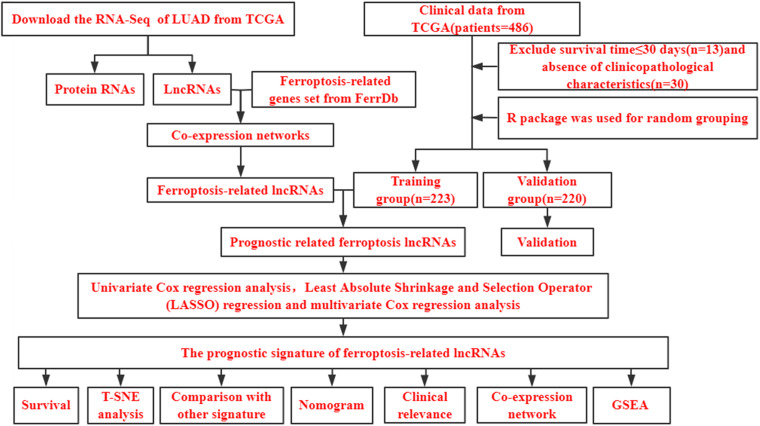
Study flow chart. LUAD, lung adenocarcinoma; TCGA, The Cancer Genome Atlas database; lncRNAs, long non-coding RNAs; T-SNE, t-distributed stochastic neighbor embedding; GSEA, gene set enrichment analysis.

Figure 1 shows a flow diagram of the study. Table 1 shows that the clinicopathological characteristics of the 443 patients with LUAD did not significantly differ between the training and validation sets. We downloaded 259 ferroptosis-related genes from FerrDb (Supplementary Table 1). To obtain high-quality lncRNA, we excluded those with a low expression level using a cutoff criterion for the average expression in all samples > 0.5. We then used the limma package in R to analyze the correlation between the expression levels of ferroptosis-related genes and lncRNA in LUAD samples and set the threshold conditions to a correlation coefficient > 0.4 and *P* < 0.001, and finally 1,138 ferroptosis-associated lncRNAs were obtained.

**TABLE 1:** Characteristics of patients with LUAD.

		**(*N*, %)**		
**Characteristics**	**Patients (*N*, %)**	**Training group (*n* = 223)**	**Validation group (*n* = 220)**	***P***
Sex				0.823
Female	244 (55.1)	124 (55.6)	120 (54.5)	
Male	199 (44.9)	99 (44.4)	100 (45.5)	
Age, years				0.190
≤ 60	148 (33.4)	81 (36.3)	67 (30.5)	
>60	295 (66.6)	142 (63.7)	153 (69.5)	
TNM stage				0.867
I	239 (54.0)	120 (53.8)	119 (54.1)	
II	107 (24.1)	56 (25.1)	51 (23.2)	
III	73 (16.5)	34 (15.3)	39 (17.7)	
IV	24 (5.4)	13 (5.8)	11 (5.0)	
pT stage				0.504
T1	152 (34.3)	82 (36.8)	70 (31.8)	
T2	234 (52.8)	110 (49.3)	124 (56.4)	
T3	38 (8.6)	20 (9.0)	18 (8.2)	
T4	19 (4.3)	11 (4.9)	8 (3.6)	
pN stage				0.570
N0	291 (65.7)	145 (65.0)	146 (66.4)	
N1	87 (19.6)	44 (19.7)	43 (19.5)	
N2	63 (14.2)	32 (14.4)	31 (14.1)	
N3	2 (0.5)	2 (0.9)	0 (0)	
pM stage				0.700
M0	419 (94.6)	210 (94.2)	209 (95.0)	
M1	24 (5.4)	13 (5.8)	11 (5.0)	
Follow-up state				0.111
Alive	290 (65.5)	138 (61.9)	152 (69.1)	
Dead	153 (34.5)	85 (38.1)	68 (30.9)	

*LUAD, lung adenocarcinoma; TNM, tumor node metastasis.*

### Development and Validation of Prognostic Ferroptosis-Related lncRNA Signature

**FIGURE 2:**
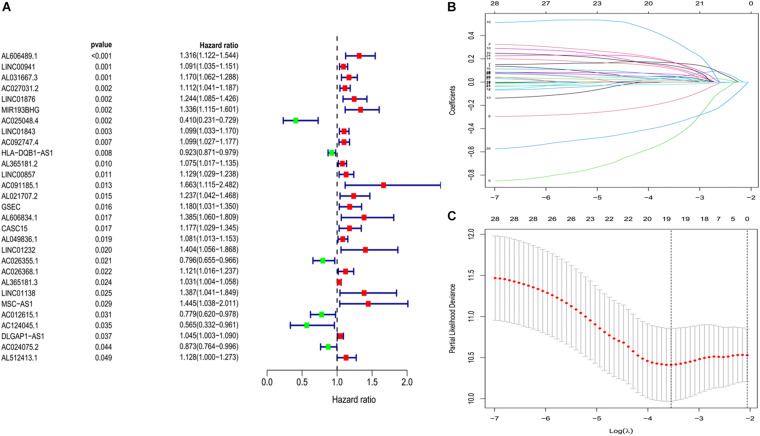
Development of prognostic ferroptosis-associated lncRNA signature. **(A)** Forest plot of univariate Cox regression identified 29 ferroptosis-related lncRNAs associated with overall survival. **(B,C)** Lasso Cox regression to identify lncRNAs associated with ferroptosis that have implications for the prognosis of LUAD. lncRNAs, long non-coding RNAs; LASSO, least absolute shrinkage and selection operator; LUAD, lung adenocarcinoma.

We constructed a ferroptosis-associated lncRNA signature based on the training group. We obtained 29 ferroptosis-associated lncRNAs associated with OS in a preliminary screen using univariate Cox analysis (*P* < 0.05; Figure 2A). We then analyzed these genes using lasso regression with 10-fold cross-validation and screened 19 ferroptosis-associated lncRNAs with a repetition rate > 900 in 1,000 replicates (Figures 2B,C). Thereafter, we conducted multivariate Cox regression analysis and constructed a lncRNA signature with the lowest AIC value. Therefore, we generated 10 ferroptosis-associated lncRNA prognostic signatures (Table 2). The median risk score classified the patients into high-risk (*n* = 111) or low-risk (*n* = 112) groups and was calculated as follows:

**TABLE 2:** Ten ferroptosis-associated lncRNAs determined *via* multivariate Cox regression analysis.

**ID**	**Coef**	**HR**	**HR.95L**	**HR.95H**	***P-*value**
LINC01843	0.0717	1.0744	1.0024	1.1515	0.0426935115489
MIR193BHG	0.3744	1.4542	1.2138	1.7422	0.000048814974
AC026355.1	–0.2992	0.7414	0.6225	0.8831	0.000798318265
AC124045.1	–0.8269	0.4374	0.2313	0.8270	0.010950731667
AC091185.1	0.8407	2.3180	1.4739	3.6457	0.000273931536
AC027031.2	0.1046	1.1103	1.0416	1.1834	0.001327246427
AL021707.2	0.1700	1.1854	0.9948	1.4124	0.057252889138
AL031667.3	0.1748	1.1909	1.0791	1.3144	0.000512851317
AL606834.1	0.3496	1.4185	1.0584	1.9012	0.019277823214
AC025048.4	–0.6308	0.5321	0.2964	0.9554	0.034615991762

*Coef, coefficient; HR, hazard ratio.*

0.0717 × Expression LINC01843 + 0.3744 × Expression MIR193BHG–0.2992 × Expression AC026355.1–0.8269 × Expression AC124045.1 + 0.8407 × Expression AC091185.1 + 0.1046 × Expression AC027031.2 + 0.1700 × Expression AL021707.2 + 0.1748 × Expression AL031667.3 + 0.3496 × Expression AL606834.1–0.6308 × Expression AC025048.4.

**FIGURE 3:**
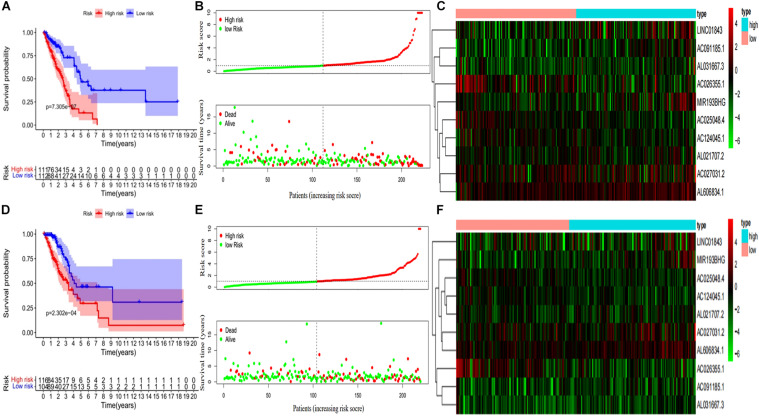
Development and validation of prognostic ferroptosis-associated long non-coding RNA signature. **(A)** Kaplan–Meier curve, **(B)** risk score and survival status, and **(C)** heat map for the training group. **(D)** Kaplan–Meier curve, **(E)** risk score and survival status, and **(F)** heat map for the validation group.

**FIGURE 4:**
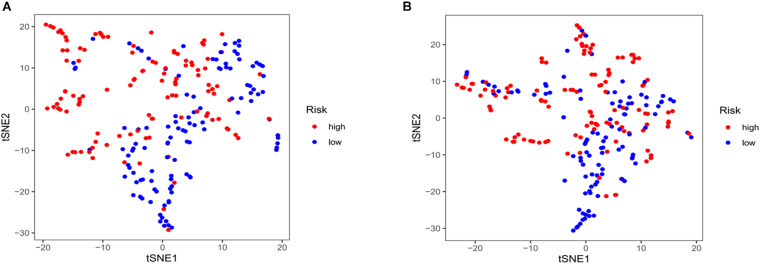
T-distributed stochastic neighbor embedding analysis. **(A)** Training and **(B)** validation sets.

Kaplan–Meier curves showed that patients with high-risk scores in the training set had a significantly higher probability of death (*P* < 0.001; Figure 3A). As risk scores increased, the risk of death increased, and survival duration decreased (Figure 3B). The risk heat map shows the expression of lncRNAs between the high- and low-risk groups (Figure 3C). We further used the Mann–Whitney *U*-test to explore the difference in lncRNA expression between the high- and low-risk groups (Supplementary Figure 1A). We then confirmed these results in the validation set, in which OS also significantly differed between the high- and low-risk groups (*P* < 0.001; Figures 3D,E). We also used the risk heat map to show the expression of lncRNA between the high- and low-risk groups in the validation set (Figure 3F) and used the Mann–Whitney *U*-test to explore the expression differences between them (Supplementary Figure 1B). The results showed that the expression of lncRNA including AC025048.4, AC026355.1, AC124045.1, AL031667.3, AL606834.1, and MIR193BHG was significantly different in the training and validation groups. We explored the distribution of the high- and low-risk groups using t-SNE (Figures 4A,B) and intuitively perceived that patients with LUAD can be better differentiated based on prognosis indicated by lncRNAs associated with ferroptosis.

### Independent Prognostic Analysis of OS

**FIGURE 5:**
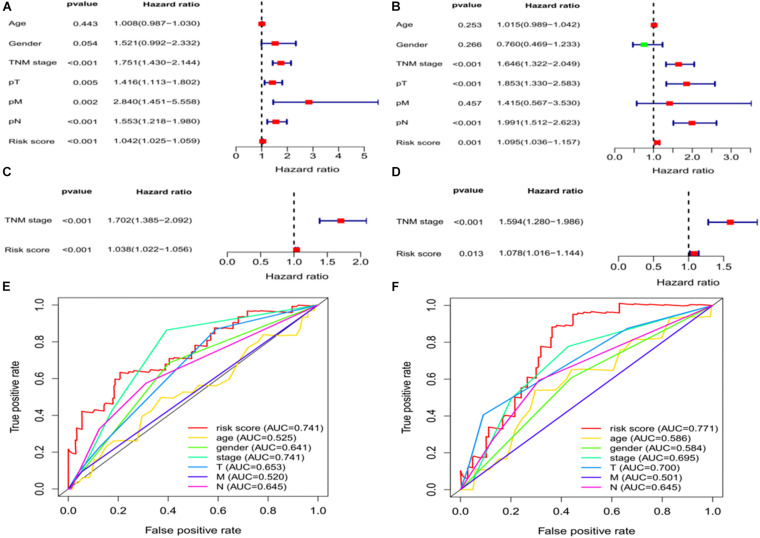
Independent prognostic factors for LUAD overall survival. Univariate Cox regression analysis in the **(A)** training and **(B)** validation sets. Multivariate Cox regression analysis in the **(C)** training and **(D)** validation sets. Receiver operating characteristic curves of risk scores and other clinical characteristics based on overall survival in the **(E)** training and **(F)** validation sets. LUAD, lung adenocarcinoma.

We assessed whether clinical characteristics (sex, age, TNM stage, pT, pN, and pM) and risk scores were independent prognostic factors for OS using univariate and multivariate Cox regression analyses. The results showed that TNM stage and risk scores were independent predictors of OS in the training and validation sets (Figures 5A–D). We further compared the diagnostic efficacy of risk scores and other baseline approaches for patients with LUAD by drawing the ROC and calculating the area under the curve (AUC). The AUC values for risk scores in the training and validation groups were 0.741 and 0.771, respectively (Figures 5E,F), which were higher than the other indicators in both groups and further confirm that ferroptosis-associated lncRNA signatures have a higher diagnostic power than other indicators in predicting the prognosis of patients with LUAD.

### Construction and Validation of the Predictive Nomogram in LUAD

**FIGURE 6:**
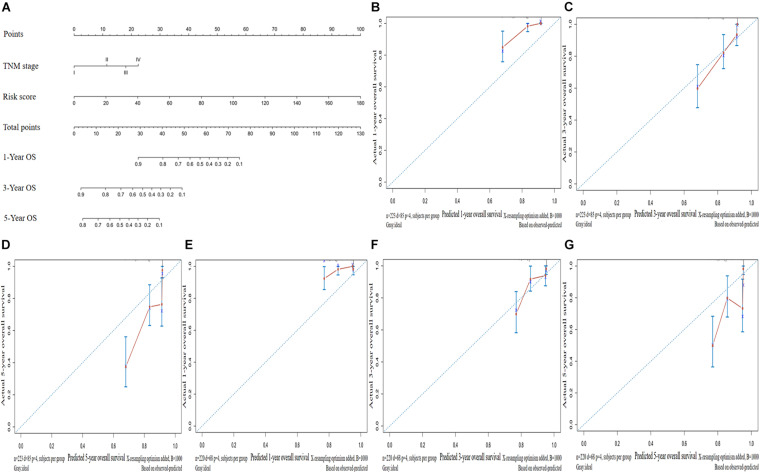
Construction and validation of a predictive nomogram. **(A)** Nomogram for predicting the overall survival (OS) of patients with LUAD at 1, 3, and 5 years. Calibration curves of nomogram for OS prediction at 1, 3, and 5 years in the **(B–D)** training and **(E–G)** validation sets. LUAD, lung adenocarcinoma.

We established a nomogram to predict the OS of patients with LUAD based on independent predictive factors derived from a multivariate Cox risk regression model (Figure 6A). The C-index of the nomogram was 0.763 [95% confidence interval (CI), 0.712–0.814)] and 0.736 (95% CI, 0.669–0.803) in the training and validation sets, respectively. The prediction model calibration curve also revealed consistent predicted and actual survival rates in the training and validation sets (Figures 6B–G).

### Correlation Between Clinicopathological Characteristics and the Expression of 10 Ferroptosis-Associated lncRNAs

**FIGURE 7:**
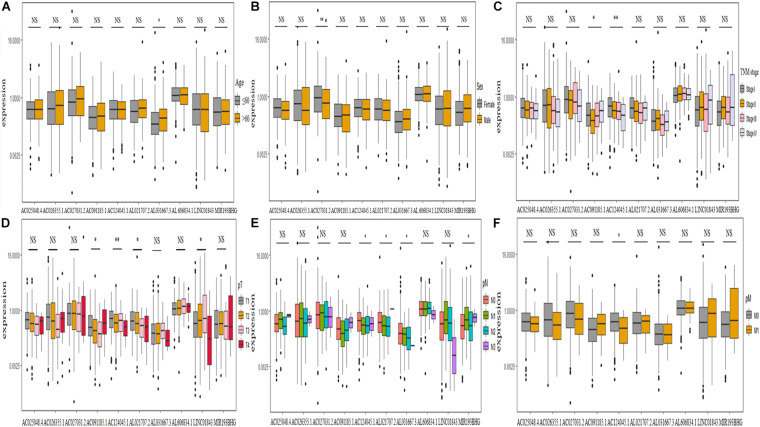
Correlation between the expression level of 10 ferroptosis-associated long non-coding RNAs and clinicopathological characteristics. **(A–F)** Age, sex, TNM stage, pT stage, pN stage, and pM stage, respectively. TNM, tumor node metastasis; NS, not significant. *P < 0.05; **P < 0.01.

We further explored the relationship between clinicopathological characteristics and found that the expression of lncRNA AL031667.3 increased with age (Figure 7A), AC027031.2 was abundantly expressed in female patients (Figure 7B), the expression of AC091185.1 and AC124045.1 was associated with TNM stage (Figure 7C), that of AC091185.1, AC124045.1, AL021707.2, and LINC01843 was associated with pT stage (Figure 7D), and that of AC124045.1, AL021707.2, AL031667.3, and MIR193BHG was associated with pN stage (Figure 7E). Patients with decreased AC124045.1 expression were more likely to have distant metastases (Figure 7F).

### Construction of Co-expression Network and GSEA

**FIGURE 8:**
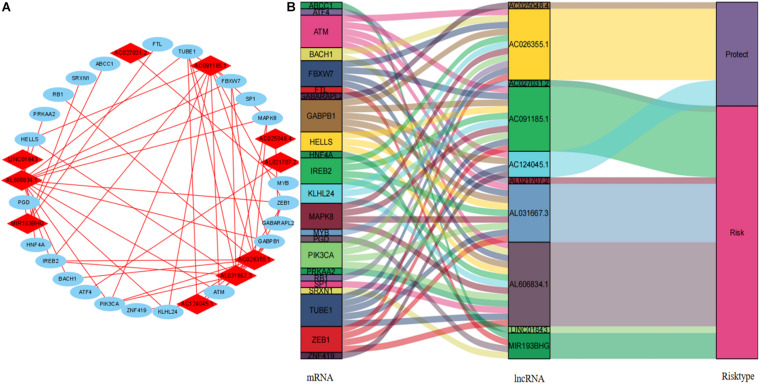
Cytoscape and Sankey diagram of the lncRNA–mRNA co-expression network. **(A)** Cytoscape map: red and blue, lncRNA and mRNA, respectively. **(B)** Sankey diagram. lncRNAs, long non-coding RNAs.

**FIGURE 9:**
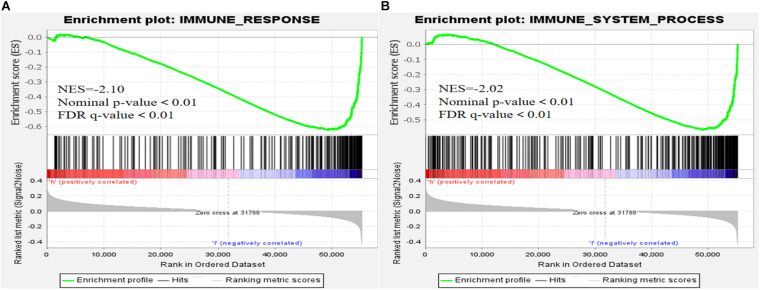
Gene set enrichment analysis. **(A)** Immune response. **(B)** Immune system process (all *P* < 0.05; false discovery rate, < 0.05).

We visualized the co-expression network between lncRNAs and mRNAs using Cytoscape (Figure 8A). We also differentiated protective and risk factors from Sankey diagrams prepared using the ggalluvial package in R (Figure 8B). The potential immune response functions of the 10 ferroptosis-associated lncRNAs were further explored by comparing immune-related GSEA enrichment between the high- and low-risk groups. The results showed that the immune response and the immune system process were significantly enriched in the low-risk group (all *P* < 0.05, FDR < 0.05; Figure 9).

## Discussion

Although screening, diagnosis, and treatment have recently progressed, LUAD remains one of the most aggressive and fatal malignancies due to its complex genetic and molecular mechanisms (Chang et al., 2021). As emerging biomarkers of genes and other molecules, lncRNAs play important roles in the occurrence and development of various tumors, including LUAD (Peng et al., 2021; Zhang L. et al., 2021). Ferroptosis is a recently established type of regulatory cell death caused by the excessive accumulation of lipid peroxides and iron-dependent reactive oxygen species (Zhang J. et al., 2021). Ferroptosis is closely associated with the pathophysiological processes of many diseases, including LUAD (Wu Y. et al., 2020). However, lncRNAs associated with ferroptosis that can determine the prognosis of patients with LUAD have remained unknown. Therefore, we aimed to construct a prognostic model by exploring lncRNAs associated with ferroptosis that could detect LUAD and improve the survival rates of patients.

We obtained 259 ferroptosis-related genes and then identified 1,138 ferroptosis-related lncRNAs through a correlation analysis. Patients with LUAD were randomized into training and validation sets. We identified 29 prognostic ferroptosis-associated lncRNAs in the training set using univariate Cox regression. We avoided overfitting using lasso regression for dimensionality reduction and obtained 19 lncRNAs that were associated with ferroptosis. We then constructed a signature comprising 10 ferroptosis-related lncRNAs with the lowest AIC values using multivariate Cox regression analysis. All patients were then assigned to high- and low-risk groups based on risk scores. The results of univariate and multivariate Cox regression analysis of the training and validation sets indicated that the risk score is an independent risk factor affecting the prognosis of patients with LUAD. The AUC and t-SNE further confirmed the accuracy and distinguishing ability of the lncRNA signature.

Nomograms are intuitive and simple prognostic prediction models that have become more prevalent in medical research and clinical practice. They have the advantages of convenient operation and high prediction accuracy, and they have been widely applied to cancer research (Chen D. et al., 2021). Since few nomograms have been constructed based on ferroptosis-associated lncRNA, we established a model based on our multivariate findings, which directly reflected the degree of influence of risk scores on predictions of patient survival. The nomogram containing risk scores discriminated the training and validation sets with a C-index of 0.763 (95% CI, 0.712–0.814) and 0.736 (95% CI, 0.669–0.803), respectively (Van Oirbeek and Lesaffre, 2010). The established calibration curve also showed that the survival rate predicted by the nomogram in the training and validation sets was consistent with the actual survival rate (Zheng et al., 2021). These results confirm the accuracy of our nomogram.

The Sankey diagram intuitively showed that seven lncRNAs were risk factors for prognosis, whereas three were prognostic protective factors. The abundant expression of LINC01843 might be associated with a poor prognosis in patients with LUAD (Li et al., 2020). MIR193BHG plays a key role in regulating physiological redox homeostasis, and lipid peroxidation is a key step in the process of ferroptosis (Wu X. et al., 2020). AC026355.1 might play a key role in the immune regulation of LUAD (You et al., 2021). We found that only AC124045.1 is associated with TNM stage, pT, pN, and pM. The expression of AC124045.1 decreased as TNM stage increased. Our results also found that AC124045.1 might be a key protective factor in terms of the prognosis of LUAD. However, little is known about this and other lncRNAs. Therefore, we plan to focus future studies on these lncRNAs to determine a new strategy for treating LUAD.

Tumor-related immune responses play important roles in cell infiltration and metastasis in the tumor microenvironment (Marar et al., 2021; Zhu et al., 2021), whereas ferroptosis and lncRNAs play key regulatory roles in tumor-related immune responses (Wang W. et al., 2019; Sun et al., 2020; Tang et al., 2020). The results of our immune-related GSEA analyses of the high- and low-risk groups revealed that the immune response and immune system processes were significantly enriched in the low-risk group, suggesting that low-risk patients have ferroptosis-related, anti-tumor immune response processes that reduce the risk of death.

Continuous breakthroughs have recently occurred regarding ferroptosis and new treatments for diseases, and novel lncRNA functions are constantly being revealed. However, many gaps in knowledge about ferroptosis and lncRNAs remain to be filled. We identified 10 ferroptosis-associated lncRNAs using high-throughput sequencing technology and elaborated a possible immune response. As far as we can ascertain, this is the first study to identify a lncRNA prognostic signal associated with ferroptosis in LUAD. However, this study has some limitations. We only used test and validation sets from TCGA database to verify the effectiveness of the lncRNA prognostic model associated with ferroptosis. Relevant basic experiments to detect the expression levels of the identified ferroptosis-associated lncRNAs in cell lines and clinical samples are scarce. Therefore, further studies are needed to clarify the mechanisms of lncRNAs associated with ferroptosis in LUAD.

## Conclusion

We identified and verified the signature of 10 lncRNAs associated with ferroptosis that have an independent prognostic value for LUAD patients and might be involved in the immune response. Therefore, we believe that our findings will serve as a potential prognostic indicator and inspire new treatment strategies involving ferroptosis to improve the prognosis of patients with LUAD.

## Data Availability Statement

The original contributions presented in the study are included in the article/Supplementary Material, further inquiries can be directed to the corresponding author/s.

## Author Contributions

DL and ZZ contributed to the conception and design. DL, ZZ, QZ, and WW analyzed and wrote the manuscript. ZZ, QZ, WW, YX, SL, and QC collected and processed the data. All the authors read and approved the final manuscript.

## Conflict of Interest

The authors declare that the research was conducted in the absence of any commercial or financial relationships that could be construed as a potential conflict of interest.

## Supplementary Material

Supplementary Figure 1 Correlation between the expression level of 10 ferroptosis-associated long non-coding RNAs and risk scores. **(A)** Training and **(B)** validation sets.

Supplementary Table 1 List of the 259 ferroptosis-related genes.
