# Supplementary figures and images for: Identification and Validation of a Ferroptosis-Related Long Non-coding RNA Signature for Predicting the Outcome of Lung Adenocarcinoma
Source: Front Genet. 2021 Jul 22;12:690509. doi: 10.3389/fgene.2021.690509 (PMC8339970; doi:10.3389/fgene.2021.690509)

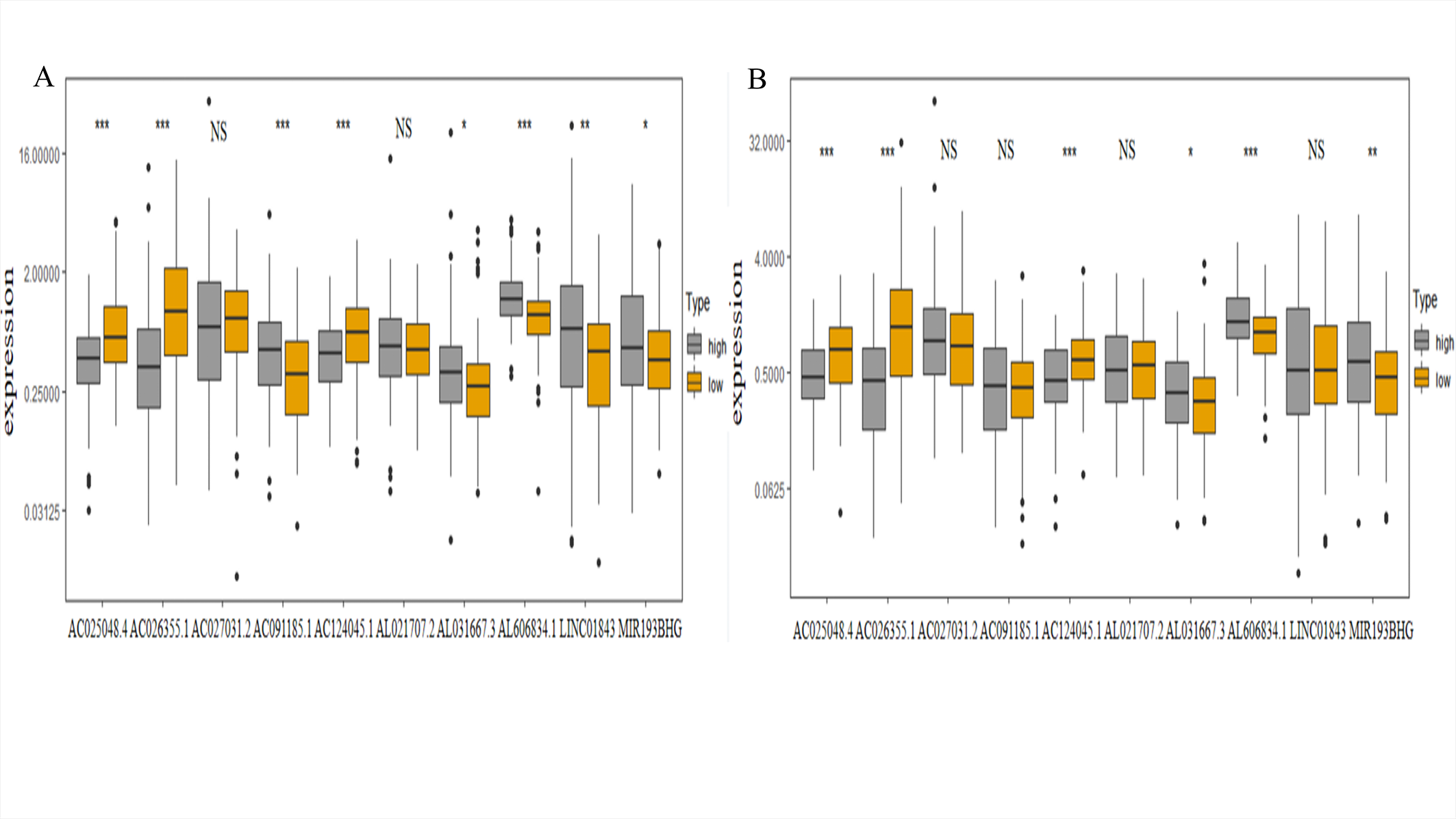

Supplement: Supplementary Figure 1 — Correlation between the expression level of 10 ferroptosis-associated long non-coding RNAs and risk scores. (A) Training and (B) validation sets. [file Image_1.TIF]
